# Liquid Biopsies for Colorectal Cancer and Advanced Adenoma Screening and Surveillance: What to Measure?

**DOI:** 10.3390/cancers15184607

**Published:** 2023-09-17

**Authors:** Ellis L. Eikenboom, Saskia M. Wilting, Teoman Deger, Malgorzata I. Srebniak, Monique Van Veghel-Plandsoen, Ruben G. Boers, Joachim B. Boers, Wilfred F. J. van IJcken, Joost H. Gribnau, Peggy Atmodimedjo, Hendrikus J. Dubbink, John W. M. Martens, Manon C. W. Spaander, Anja Wagner

**Affiliations:** 1 Department of Clinical Genetics, Erasmus MC Cancer Institute, University Medical Center Rotterdam, 3000 CA Rotterdam, The Netherlands; e.eikenboom@erasmusmc.nl (E.L.E.); m.srebniak@erasmusmc.nl (M.I.S.); m.plandsoen@erasmusmc.nl (M.V.V.-P.); 2 Department of Gastroenterology & Hepatology, Erasmus MC Cancer Institute, University Medical Center Rotterdam, 3000 CA Rotterdam, The Netherlands; v.spaander@erasmusmc.nl; 3 Department of Medical Oncology, Erasmus MC Cancer Institute, University Medical Center Rotterdam, 3000 CA Rotterdam, The Netherlands; s.wilting@erasmusmc.nl (S.M.W.); t.deger@erasmusmc.nl (T.D.); j.martens@erasmusmc.nl (J.W.M.M.); 4 Department of Developmental Biology, Erasmus University Medical Center, 3000 CA Rotterdam, The Netherlands; r.g.boers@erasmusmc.nl (R.G.B.); j.b.boers@erasmusmc.nl (J.B.B.); j.gribnau@erasmusmc.nl (J.H.G.); 5 Center for Biomics, Erasmus Medical Center, 3000 CA Rotterdam, The Netherlands; w.vanijcken@erasmusmc.nl; 6 Department of Pathology, Erasmus MC Cancer Institute, University Medical Center Rotterdam, 3000 CA Rotterdam, The Netherlands; p.atmodimedjo@erasmusmc.nl (P.A.); h.dubbink@erasmusmc.nl (H.J.D.)

**Keywords:** cfDNA, ctDNA, genome-wide methylation, Lynch syndrome, advanced adenoma, screening

## Abstract

**Simple Summary:**

Colonoscopies are effective in the prevention of colorectal cancer (CRC) but considered burdensome. An alternative for or addition to this procedure might be found in circulating tumor DNA (ctDNA) analysis. However, to date, it is not clear which analysis is most suitable for such a ctDNA-based blood test for CRC. Therefore, we assessed this in ten patients with colonoscopies for Lynch syndrome or in the context of the Dutch national screening program who were diagnosed with CRC or its precursor lesion (advanced adenoma). The results of this proof-of-principle study could form the foundation for subsequent studies on ctDNA-based blood test development for CRC screening and management, specifically in carriers of Lynch syndrome.

**Abstract:**

Colorectal cancer (CRC) colonoscopic surveillance is effective but burdensome. Circulating tumor DNA (ctDNA) analysis has emerged as a promising, minimally invasive tool for disease detection and management. Here, we assessed which ctDNA assay might be most suitable for a ctDNA-based CRC screening/surveillance blood test. In this prospective, proof-of-concept study, patients with colonoscopies for Lynch surveillance or the National Colorectal Cancer screening program were included between 7 July 2019 and 3 June 2022. Blood was drawn, and if advanced neoplasia (adenoma with villous component, high-grade dysplasia, ≥10 mm, or CRC) was detected, it was analyzed for chromosomal copy number variations, single nucleotide variants, and genome-wide methylation (MeD-seq). Outcomes were compared with corresponding patients’ tissues and the MeD-seq results of healthy blood donors. Two Lynch carriers and eight screening program patients were included: five with CRC and five with advanced adenomas. cfDNA showed copy number variations and single nucleotide variants in one patient with CRC and liver metastases. Eight patients analyzed with MeD-seq showed clustering of Lynch-associated and sporadic microsatellite instable lesions separate from microsatellite stable lesions, as did healthy blood donors. In conclusion, whereas copy number changes and single nucleotide variants were only detected in one patient, cfDNA methylation profiles could discriminate all microsatellite instable advanced neoplasia, rendering this tool particularly promising for LS surveillance. Larger studies are warranted to validate these findings.

## 1. Introduction

Colorectal cancer (CRC) is the third-leading cause of cancer-related death worldwide [1]. For this purpose, an increasing number of developed countries implemented dedicated CRC screening programs. The Dutch National Colorectal Cancer screening program (SP) was initiated in 2014 and invites all Dutch inhabitants aged 55–75 biennially for a fecal immunohistochemical test (iFOBT), which is followed by colonoscopy in cases of positive results. This way of screening proved to be effective in detecting CRC and its precursor lesions, advanced adenoma (AA), and therefore, it contributes to a decrease in CRC incidence and mortality by detecting CRC earlier [2].

Besides this nationwide program, additional dedicated screening programs exist for individuals who are at high risk of developing CRC, such as carriers of a pathogenic variant in one of the DNA mismatch repair (MMR) genes, resulting in Lynch Syndrome (LS) [3,4,5,6,7]. LS carriers have up to a 55% chance of developing CRC during their lives, but exact risk estimates depend on the specific MMR gene involved [8,9,10]. A hallmark of LS-associated tumors is microsatellite instability (MSI-H), although this is also detected in a minority of sporadic CRCs [11,12]. To prevent CRC development, LS carriers are advised to undergo biennial colonoscopy [13]. This surveillance strategy proved to be effective in diminishing CRC-related deaths in LS carriers [14].

Despite its high efficiency, the iFOBT has a low sensitivity. Nearly half of the people who are advised to have a colonoscopy do not have colorectal anomalies after all. Similarly, the majority of LS carriers do not have CRC or AA during biennial colonoscopic surveillance. Additionally, colonoscopies are burdensome. Therefore, new, less invasive strategies for early CRC detection are warranted.

Cell-free DNA (cfDNA)-based blood tests seem to be particularly promising for this purpose [15]. cfDNA is shed into the circulation by dying cells and can thus also contain a fraction of circulating tumor DNA (ctDNA) [16]. For ctDNA detection in the total pool of cfDNA, a myriad of tests can be performed, including the assessment of single nucleotide variants (SNVs), chromosomal copy number variations (CNVs), microsatellite instability (MSI), and DNA methylation, or a combination of all the above, and ctDNA is often determined via a multitude of assays currently applied in various settings [17]. For example, tumor-specific SNV or methylation patterns detected in ctDNA correlate with prognosis [18,19] and can be used for disease monitoring [20,21,22], while genomic copy number instability (CNI) scores based on CNVs detected in the ctDNA are helpful in determining more tailored treatment strategies [23]. Theoretically, ctDNA analyses can also be used for cancer diagnosis; however, generally, more ctDNA is shed into the circulation in more advanced stages [24,25,26], indicating that a highly sensitive assay is required for this purpose. In addition, even though SNVs and short indels in cfDNA can be detected with high sensitivity and are predictive of CRC recurrence, these should ideally be known upfront to develop a patient-specific screening test and are thus less useful for primary cancer screening.

Ideally, cfDNA could even be used for the detection of precursor lesions to prevent cancer outgrowth and for locating the origin of this lesion. This is particularly of importance to LS carriers and other individuals with a hereditary predisposition to develop cancer. Specifically, cfDNA methylation profiles seem to be promising for this purpose. Previous research showed clustering of AA separate from healthy blood donors based on 11 methylation markers [27]. More insight into genome-wide methylation patterns may provide us with an alternative method for both colorectal and extra-colonic surveillance in hereditary predisposed patients. Conventional genome-wide methylation analyses are, however, extremely expensive at this moment and require high amounts of input DNA.

Recently, we presented a cheaper alternative to genome-wide methylation analysis (MeD-seq) [28] and showed that this method can also reliably be applied to lower amounts of cfDNA [29]. Therefore, we aimed to assess which assay or combination of assays would be most promising for the development of a cfDNA-based blood test (MeD-seq, CNV, and SNV analyses) to promptly detect AA and CRC in the context of the nationwide CRC screening program and LS surveillance.

## 2. Materials and Methods

For this proof-of-concept study, we approached participants of the National CRC screening program with a positive iFOBT, as well as molecularly proven LS carriers, with a planned colonoscopy in the Erasmus MC, Rotterdam (The Netherlands) between 7 July 2019 and 3 June 2022. The Erasmus MC Institutional Review Board approved this study (NL68955.078.19). Additionally, this study was reported in the International Clinical Trials Registry Platform (NL8695). The study procedures were performed as described in the Declaration of Helsinki. Written informed consent was obtained from every participant. Due to the explorative nature of this study, no sample size calculation was performed. The results of this study are intended as the foundation for a larger follow-up study.

### 2.1. Patients

Potential participants were informed about the study and the theoretical chance of finding unexpected findings. Upon informed consent, blood was drawn just before the colonoscopy was carried out via peripheral venous catheter, which is required for colonoscopy sedation; first, blood was collected in one mock EDTA tube, and subsequently, it was collected in two Streck Cell-Free BCT tubes (La Vista, NE, USA) [30]. The tubes were kept at room temperature and transferred to the laboratory as soon as possible (after a maximum of four days) for plasma isolation, as recommended by the manufacturer’s instructions. Patients with AA (adenomas ≥ 10 mm in size with a villous component or high-grade dysplasia) or CRC (advanced neoplasia) identified in the colonoscopy were analyzed. CRCs were routinely tested for the presence of MSI-H as the standard of care. However, some adenomas were stained for the presence of DNA mismatch repair proteins (MMR IHC). For readability, only the terms MSI-H and microsatellite-stable were used. Lesions with all four DNA MMR proteins present were classified as MSI-stable, whereas the absence of at least one DNA MMR protein was classified as MSI-H.

### 2.2. Plasma and cfDNA Handling

Plasma isolation was performed as described previously [31]. Plasma samples were stored in up to six 2 mL vials per participant at −80 °C. Only in cases in which participants were diagnosed with an advanced adenoma or CRC, the participants were included for cfDNA analyses. The cfDNA of these patients was isolated from stored plasma, as described previously, using the QIAamp Circulating Nucleic Acid Kit according to the manufacturer’s instructions (Qiagen, Hilden, Germany) [29]. the obtained cfDNA was analyzed for CNVs, tumor-specific SNVs, and genome-wide methylation profiles.

### 2.3. CNV, SNV, and MeD-Seq Analyses

#### 2.3.1. CNVs

To assess the presence of CNVs in the cfDNA, an automated NGS workflow was performed using the VeriSeq (Illumina, Cambridge, UK) and the Microlab Star Robot (Hamilton, Gräfelfing, Germany) according to the manufacturer’s instructions, as described before [31]. In brief, 1 mL of plasma was used for analyses. A unique synthetic DNA ‘barcode’ (index) was attached to each sample, and the library product was quantified using a fluorescent dye and compared to a DNA standard curve. Lastly, shallow whole genome sequencing was performed on a NextSeq500 sequencer, yielding 2 × 36 paired-end reads in a 48-plex reaction. The SeqFF model was used as a surrogate marker to assess the percentage of short fragmented cfDNA that was likely of tumor origin (ctDNA) in the total cfDNA pool (herein called the percentage tumor cfDNA) [32]. Additionally, ctDNA in the pool of cfDNA was estimated using ichorCNA software version 0.2.0 [33]. Outcomes were analyzed with WISECONDOR statistical software version 2.0.1 [34]. Next, cfDNA fragment sizes were assessed.

#### 2.3.2. SNVs

To assess whether SNVs were present in the cfDNA, 10 ng cfDNA was analyzed with the Next Generation Sequencing (NGS) OncomineTM Colon cfDNA Assay, as described previously, using variantCaller version v5.10.0 in cases of at least three or more unique molecules [35]. Sequencing for diagnostic purposes aimed for at least 25,000 reads. This panel covers the following genes with >240 hotspots: AKT1, APC, BRAF, CTNNB1, EGFR, ERBB2, FBXW7, GNAS, KRAS, MAP2K1, NRAS, PIK3CA, SMAD4, and TP53.

Formalin-fixed paraffin-embedded (FFPE) tissue, obtained as the standard of care, was retrieved for patients included in this study. The tissue was analyzed by means of NGS analysis to assess the presence of tumor-specific SNVs using the Ion Torrent S5 sequencing system (ThermoFisher, Waltham, MA, USA). For this, the Ion Ampliseq Library Plus Kit 2.0 was used, with a custom-made primer panel including the same 14 genes as the cfDNA Assay panel [36]. Templates were prepared using the Ion Chef with the 540 Chef kit. Data were analyzed using variantCaller v5.10.0, aiming for 1250 reads.

#### 2.3.3. MeD-Seq

Methylation patterns in cfDNA were analyzed by MeD-seq, as described previously [28]. In short, MeD-seq uses a methylation-dependent restriction enzyme (LpnPI) to generate 32 base-pair-long (methylated) DNA fragments for sequencing. For MeD-seq analyses, up to 25 ng of input DNA was used. Input DNA was end-repaired and ligated to dual-indexed adaptors. The resulting libraries were sequenced on the Illumina HiSeq 2500 sequencer, yielding single read 50 base pair reads. All patients were analyzed with MeD-seq, except for CATCA006 and CATCA044, as there was insufficient cfDNA remaining for MeD-seq analysis. The MeD-seq results were compared to those of healthy blood donors (HBDs).

#### 2.3.4. MeD-Seq Data Processing and Analysis

Subsequent data processing was carried out as previously described and included a filtering step based on LpnPI restriction site occurrence between 13 and 17 bp from the 5’ or 3’ end of the read, after which only reads originating from methylated DNA fragments remained [28,29]. Using all unambiguously mapped reads, count scores were assigned to each individual LpnPI site in the human genome. Subsequently, count scores for individual CpG sites were summarized into 2 kilobase (kb) regions surrounding all known transcription start sites (TSS) annotated in ENSEMBL. Only regions containing data in at least 50% of all samples were included in subsequent analyses, resulting in a total of 41,570 regions on chromosomes 1–22. Data were normalized to the total number of reads passing the LpnPI filter per sample, after which square root transformation was applied to reduce skewness in the data distribution.

Differentially methylated regions (DMRs) between patients and HBDs were identified using LIMMA, resulting in 2798 regions [37].

Principal component analysis (PCA) was performed using the singular value decomposition function (svd) in the R base package (the R project for statistical computing) on mean-centered MeD-seq values from either the most variable regions (for example, regions showing a standard deviation over all samples that is larger than the median standard deviation observed for all regions) or the DMRs. The resulting principal components were uncorrelated summary statistics, each reflecting the methylation data for a mixture of correlated genomic regions. To generate an overall score for aberrant cfDNA methylation per patient, Z-scores were calculated per region for every patient relative to our normal control panel of 12 HBDs. These Z-scores per region were squared and summed into a methylation score, as described before [28,29].

## 3. Results

### 3.1. Patients

Of the 211 patients willing to participate, ten patients were diagnosed with AA or CRC and were thus included in this study (Table 1 and Table S1). Of these patients, eight participated in the Dutch national CRC screening program and were referred to the Erasmus MC for a colonoscopy after a positive iFOBT. Two patients were LS carriers with a biennial surveillance colonoscopy (CATCA038 and CATCA099). Of these patients, five presented with CRC (with stages ranging from TxN0M0 to T3NxM1). One of the CRCs (CATCA008) was found to be MSI-H due to sporadic *MLH1* promoter hypermethylation; the other four CRCs were found to be microsatellite-stable. The remaining five patients presented with AA; one of them turned out to be MSI-H (LS carrier).

### 3.2. Single Nucleotide Variants (SNVs) in Tissue and Corresponding Plasma cfDNA

Tumor tissue from all included patients harbored at least one mutation covered by the cfDNA Oncomine panel (Table 1 and Table 2). Two patients showed abnormal SNVs in the plasma cfDNA. In patient CATCA016, who presented with metastasized CRC, concordance was observed between tissue and cfDNA for two somatic variants (Table 1). Patient CATCA008 presented with an SNV in the cfDNA that was not present in the tissue, despite being covered in both sequencing panels. Since only five molecules were detected, this might have been an artifact. Alternatively, this observation could be due to tumor heterogeneity, clonal hematopoiesis, or sampling error [38,39]. In the remaining six patients, no alterations were detected in cfDNA.

### 3.3. cfDNA CNV and Fragment Size Analysis

Unbalanced chromosome aberrations were convincingly detected in CATCA016 but not in any of the other patients (Supplementary Figure S1). For an overview of the CNVs, please see Table 3. Of note, cfDNA fragment size comparisons in patients with CRC or AA were hampered by the small sample size and low cfDNA levels, but they were not found to be smaller than in healthy controls (Figure S2). Nevertheless, the quantile 5% analyses showed a slightly elevated level of insert size < 130 bp in the AA group in comparison to controls (Figure S2B).

### 3.4. Genome-Wide cfDNA Methylation Profiling

For eight patients, plasma was subjected to MeD-seq; for the remaining two patients, insufficient cfDNA was left to perform this analysis. Genome-wide cfDNA methylation profiles were successfully generated for these patients as well as for 12 healthy blood donors (HBDs). Exploratory unsupervised analyses showed a clear distinction between the two LS-associated AA and the sporadic MSI-H CRC on the one hand and the MSS lesions and HBDs on the other hand (Figure S3).

**Figure 1:**
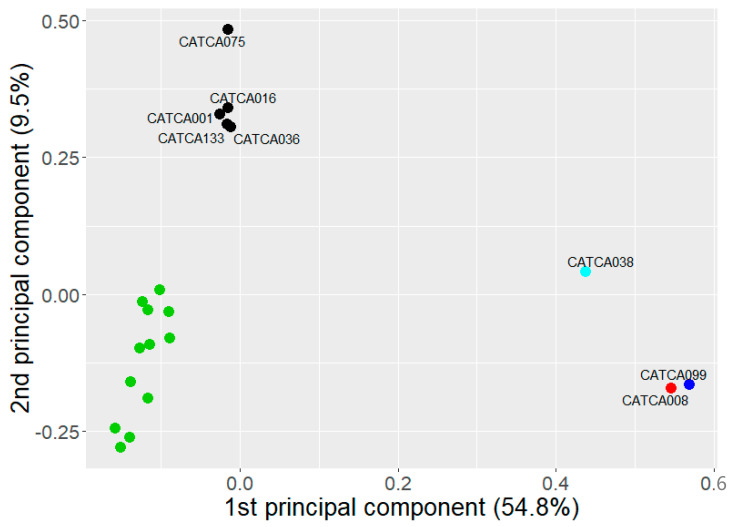
Principal component analysis (PCA) for patients included in our study and healthy blood donors (HBDs, in green) based on the DMRs, with on each axis the percentage of variation that can be explained by that specific principal component. The patient with MSI-H CRC and LS carriers with AA are depicted in red and blue, respectively, while sporadic MSS lesions are shown in black.

Using a supervised approach in which we only included differentially methylated regions between the eight patients included in our study on the one hand and HBDs on the other hand (2798 DMRs with an unadjusted *p*-value of <0.05 in LIMMA analysis, Table S2), we observed a clear separation between LS-associated and/or MSI-H lesions, versus MSS lesions versus HBDs (Figure 1).

**Figure 2:**
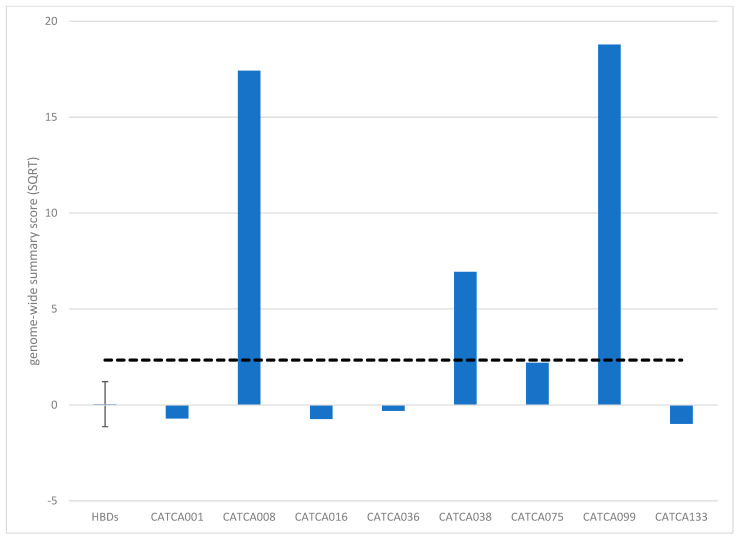
Summary methylation scores upon genome-wide methylation analysis (MeD-seq) based on the entire genome (genome-wide summary score). Genome-wide summary scores above 2.34 were considered elevated (dashed line).

To facilitate the use of this assay for screening or surveillance, we need to capture the methylation profile in a single score. For this purpose, we calculated a summary methylation score based on either the entire genome (genome-wide summary score) or the 2798 DMRs between patients and HBDs (DMR summary score; see the methods section for more information about this calculation). The resulting scores are shown in Figure 2 and Table S3. Genome-wide summary scores above 2.34 and DMR summary scores above 2.25 were considered elevated, which represented the upper 95% CI bound of the HBDs. As expected from the results above, the genome-wide summary score only detected the LS-associated AA and sporadic MSI-H CRC, whereas the DMR summary score was elevated in seven out of eight patients (Figure 2, Table S3).

## 4. Discussion

In this proof-of-principle study, we assessed to what extent different cfDNA analysis methods could be used in screening and surveillance for AA and CRC. SNVs and CNVs were only detected in the plasma of one patient presenting with liver metastases at diagnosis. By contrast, genome-wide cfDNA methylation profiling (MeD-seq) followed by supervised data analysis could distinguish advanced neoplasia samples from HBDs. This suggests that genome-wide cfDNA methylation profiling could sensitively reflect tumor-specific anomalies, and as such, it may represent a promising and minimally invasive tool for CRC screening and surveillance. Interestingly, both LS-associated AA and the sporadic MSI-H CRC showed very distinct cfDNA methylation profiles, particularly highlighting the potential of cfDNA methylation profiling for LS surveillance.

### 4.1. SNVs

We did not find tumor-specific SNVs in cfDNA that were present in matched tumor tissue, except for one patient with metastasized CRC. This is in line with previous studies, which have also found that ctDNA levels increase with the CRC stage [33,40]. Even though we performed SNV analyses using a sensitive and dedicated CRC panel including unique molecular identifiers, we could not trace these SNVs in the blood, except for the patient with metastasized CRC. As SNVs were detected in the matched primary FFPE tissue of all patients included, the lack of detection in the plasma is most likely due to the extremely low levels of ctDNA present in the blood of early-stage CRCs and AA. The detection of a larger number of SNVs in a single patient is expected to increase the sensitivity, but it requires prior knowledge of the molecular characteristics of the tumor. This is followed by the design of patient-specific assays, which renders this approach infeasible for general CRC screening and high-risk population surveillance.

### 4.2. CNVs

Results similar to SNVs were observed for CNVs: in only one patient, an abnormal CNV pattern was identified. The literature indicates reliable CNV analysis in plasma requires at least 5% ctDNA within the total pool of cfDNA, which may explain our results [41]. According to our data, the CNV cfDNA analyses using our current approach do not seem to be a suitable option for CRC screening or surveillance. However, more sensitive methods have emerged in the field of oncology to assess cfDNA CNVs in the last few years; with so-called fragmentomics analyses, one specifically focuses on cfDNA fragments of certain sizes to more closely assess ctDNA [42,43]. Due to the limited sample size, we were unable to assess the added value of fragmentomics in the current dataset. Nevertheless, the quantile 5% analyses showed a slightly elevated level of insert size < 130 bp in the AA group in comparison with controls. Although the current results do not support CNV analysis of cfDNA as a promising approach for CRC screening and surveillance of high-risk individuals, bioinformatics analyses are continuously being improved, possibly enabling more sensitive CNV analyses in cfDNA in future research.

### 4.3. Genome-Wide Methylation

In contrast to the other assays performed, genome-wide methylation profiling by MeD-seq on blood plasma-derived cfDNA discriminated MSS advanced neoplasia samples from both MSI-H and LS-driven neoplasia and HBDs. This is in line with the known differences in phenotype between MSS and MSI-driven CRC lesions (chromosomal instability, CIN, versus CpG island methylator phenotype, CIMP, often accompanied by MSI-H) [44,45,46]. Larger studies should be performed to assess to what extent this holds true and to determine and validate the most informative DMRs.

### 4.4. Application of MeD-Seq in Lynch Syndrome Surveillance

Interestingly, we found that MSI-H - and LS-associated advanced neoplasia clustered together based on their methylation profiles. Of note, even the advanced adenoma in the LS carrier clustered more toward the sporadic and LS-associated MSI-H lesions, despite still being microsatellite-stable. Therefore, this tool might be particularly promising in LS CRC surveillance, although larger studies are needed to validate the performance of this assay in larger groups of LS patients, and sporadic microsatellite stable and MSI-H lesions. MeD-seq cfDNA profiling might contribute to the decision of who needs further colonoscopic assessment in the context of LS colorectal cancer surveillance. Additionally, we need to explore whether extra-colonic LS-associated tumors can also be detected by MeD-seq to establish whether cfDNA methylation profiling represents a promising tool to use in surveillance for all LS-associated tumors. Previous research pointed out that methylation profiles contain information about the tissue of origin, enabling further work-up and treatment [47]. In this case, all surveillance for LS carriers could be performed by biennial cfDNA methylation assessment.

### 4.5. Limitations

Our study has several limitations. First, due to the proof-of-principle nature of this study, we had a small number of patients with CRC or AA with a mixed etiology (LS versus sporadic). Due to the small and heterogeneous patient group, we do not know to what extent the DMRs identified here are representative of the different populations of interest.

Future studies focusing on the added value of cfDNA surveillance in LS patients should consist of a multicenter approach to increase the number of patients. Second, because our study explored multiple techniques, for two patients, we unfortunately had too little material to perform all envisioned assays. On the other hand, this underlines the need for assays requiring a limited amount of input while generating a rich data output (for example combining methylation with SNVs and/or CNVs) [48,49]. Third, CNVs and methylation were not assessed in these patients’ tissues. However, previous research showed that for a selection of genes associated with metastasized CRC, the profiles matched the tumor tissue [29]. Last, although HBDs were obtained from the healthy population, no information was available on iFOBT results and/or recent colonoscopy, meaning we cannot rule out the presence of advanced neoplasia without complaints in these individuals.

## 5. Conclusions

In conclusion, this proof-of-principle study highlights the promise of cfDNA methylation profiling, specifically in the context of LS surveillance, and provides a starting point for future research in this area.

## Figures and Tables

**Table 1:** Baseline characteristics of analyzed patients and overview of analyses performed.

ID	Lesion Characteristics ^╪,#^	Concent-Ration cfDNA [ng/µL]	Tumor Fraction in cfDNA Pool ^¥^	cfDNA CNVs	SNVs in Tissue	cfDNA SNV	DMR Summary Score ^∆^
CATCA001	TxN0M0 CRC, MSI-stable	1.16	Unknown	−	+	−	↑
CATCA006	AA, MSI-stable	2.13	0.008897	−	+	−	N/A
CATCA008	pT2N0 CRC, MSI-H	1.84	0.008029	−	+	Disc	↑
CATCA016	T3NxM1 CRC, MSI-stable	3.08	0.0488	+ ^1^	+	Conc	↑
CATCA036	AA MSI-stable	1.85	0.00427	−	+	−	↑
CATCA038	AA, MSI-H	1.64	0.006391	−	+	−	↑
CATCA044	pT3N2bM0, MSI-stable	2.29	0.01411	−	+	−	N/A
CATCA075	AA, MSI-stable	1.74	0.005006	−	+	−	↑
CATCA099	AA, MSI-stable	1.89	0.00988	−	+	−	↑
CATCA133	pT1 CRC, MSS	1.55	0.01028	−	+	−	Normal

AA = advanced adenoma, CRC = colorectal cancer; conc = concordant; disc = discordant; MSI-H = high microsatellite instability; N/A = not applicable; − = negative; + = positive; ↑ = elevated. ^1^ Complex abnormal pattern. ^#^ Tumors tested with immunohistochemistry not lacking staining of at least one of the DNA MMR proteins were scored as MSS. ^∆^ DMR summary score shows methylation scores upon genome-wide methylation (MeD-seq) analysis based on the entire genome (genome-wide summary score) of the 2798 differentially methylated regions (DMRs) between patients included in our study and healthy blood donors. ^¥^ Estimated by ichorCNA software. ^╪^ In cases of colorectal carcinoma, TNM stage is listed, in cases of advanced adenoma, the type of advanced adenoma, size, and grade of dysplasia are listed.

**Table 2:** Outcomes of Next-Generation Sequencing (NGS) panels on tissue and cfDNA isolated from blood plasma in each patient.

ID	Lesion	Tissue, Variants Detected	VAF	In cfDNA Panel?	cfDNA Variants Detected	VAF
CATCA001	CRC	KRAS c.38G>A; p.G13D APC c.4470_4479del; p.H1490Qfs*14	68% 26%	Y Y	None	N/A
CATCA006	AA	APC c.4187_4188del; p.F1396* KRAS c.35G>A; p.G12D KRAS c.35G>T; p.G12V	39% 14% 24%	N Y Y	None	N/A
CATCA008	CRC	BRAF c.1799T>A; p.V600E POLD1 c.730T>C; p.Y244H PTEN c.517C>T; p.R173C RNF43 c.816_817del; p.A273Hfs*8 RNF43 c.1976del; p.G659Vfs*41 STK11 c.998G>A; p.R333H TP53 c.758C>T; p.T253I	38% 26% 42% 9% 40% 40% 43%	Y N N N N N Y	APC c.4387dupA:p.K1462fs ^∆^	0.36%
CATCA016	CRC	KRAS c.436G>A; p.A146T TP53 c.844C>T; p.R282W APC c.3859del; p.I1287*	72% 83% 44%	Y Y N	KRAS p.A146T TP53 p.R282W GNAS p.R201H	0.48% 0.95% 0.24%
CATCA036	AA	APC c.4476del; p. T1493Rfs*14	35%	Y	None	N/A
CATCA038	AA	FBXW7 c.1528G>A; p.D510N KEAP1 c.992C>T; p.A331V RNF43 c.450+2T>C; p.? STK11 c.435G>T; p.E145D	32% 45% 41% 16%	Y N N N	None	N/A
CATCA044	CRC	APC c.4058_4075delinsCG; p.E1353Afs*57 TP53 c.635_636del; p.F212Sfs*3 TP53 c.701del; p.Y234Sfs*13	64% 39% 38%	Y Y Y	None	N/A
CATCA075	AA	APC c.3927_3931del; p.E1309Dfs*4 KRAS c.35G>A; p.G12D	26% 13%	Y Y	None	N/A
CATCA099	AA	APC c.3948_3949del; p.E1317Rfs*14 TP53 c.388C>T; p.L130F KRAS c.35G>A; p.G12D TP53 c.743G>A; p.R248Q	79% 44% 9.2% 8.8%	Y N Y Y	None	N/A
CATCA133	CRC	APC c.4199C>A; p.S1400* FBXW7 c.1513C>T; p.R505C KRAS c.34G>C; p.G12R KRAS c.35G>A; p.G12D (in trans) MTOR c.4444C>T; p.R1482C TP53 c.659A>G; p.Y220C TP53 c.733G>A; p.G245S TP53 c.743G>A; p.R248Q	51% 48% 10% 2% 13% 14% 30% 36%	Y Y Y Y N Y Y Y	None	N/A

AA = advanced adenoma; CRC = colorectal cancer; N = no; N/A = not applicable; VAF = variant allele frequency; Y = yes, ^∆^ No clues for homozygous deletion of this gene in tissue.

**Table 3:** Copy number variations (CNVs) detected in cfDNA, isolated from blood.

Sample ID	Lesion	Percentage of Short Fragmented cfDNA ^∆^	Tumor Fraction in cfDNA Pool ^¥^	Number of Reads (mln) Used for Wisecondor Analysis	cfDNA CNV Wisecondor Profile
CATCA001	CRC	2.6%	Unknown	24.4	no CNVs detected
CATCA006	AA	7%	0.008897	17.4	no CNVs detected
CATCA008	CRC	2.4%	0.008029	20	no CNVs detected
CATCA016	CRC	5.4%	0.0488	20	Complex abnormal pattern: 1p loss, 5q loss, trisomy 7, trisomy 9, 12p gain, trisomy 13, 17p loss, 18q loss, 20q gain, 21q loss (Figure S1)
CATCA036	AA	4%	0.00427	15.7	no CNVs detected
CATCA038	AA	2.8%	0.006391	16.2	no CNVs detected
CATCA044	CRC	2.3%	0.01411	92	no CNVs detected
CATCA075	AA	6.3%	0.005006	16.1	no CNVs detected
CATCA099	AA	2.9%	0.00988	13.5	no CNVs detected
CATCA133	CRC	2.3%	0.01028	16.1	no CNVs detected

AA = advanced adenoma; CRC = colorectal carcinoma; CNV = copy number variations. ^¥^ Estimated by IchorCNA. ^∆^ Estimated by SeqFF model.

## Data Availability

All data generated or analyzed during this study are included in this published article and its supplementary information files.

## Supplementary Materials

The following supporting information can be downloaded at: https://www.mdpi.com/article/10.3390/cancers15184607/s1, Figure S1: cfDNA CNV Wisecondor profile of patient CATCA016. This patient had colorectal cancer with liver metastases; Figure S2: (A) Median insert sizes and (B) quantile 5% insert sizes in cfDNA of patients with advanced adenoma (green), colorectal cancer (blue), and controls (red); Figure S3: Principal component analysis (PCA) of patient samples analyzed with genome-wide methylation analysis (MeD-seq) for patients included in our study and healthy blood donors (HBDs, in green); Table S1: Patient characteristics of patients included for cell-free DNA analyses; Table S2: List of differentially methylated regions; Table S3: Summary methylation scores upon genome-wide methylation analysis (MeD-seq).

## Abbreviations

AA	advanced adenoma
AN	advanced neoplasia
CNV	copy number variation
CRC	colorectal cancer
ctDNA	circulating tumor DNA
DMR	differentially methylated region
FFPE	Formalin-fixed paraffin-embedded
HBD	healthy blood donor
HGD	high-grade dysplasia
IFOBT	fecal immunohistochemical test
LS	Lynch syndrome
MeD-seq	genome-wide methylation
MMR	mismatch repair
MMRp	mismatch repair proficient
MSI-H	microsatellite instability-high
MSS	microsatellite stable
SNV	single nucleotide variant
SP	national colorectal cancer screening program
TVA	tubulovillous adenoma
VAF	variant allele frequency
