# Supplementary material for: Liquid Biopsies for Colorectal Cancer and Advanced Adenoma Screening and Surveillance: What to Measure?
Source: Cancers (Basel). 2023 Sep 17;15(18):4607. doi: 10.3390/cancers15184607 (PMC10526371; doi:10.3390/cancers15184607)
Supplement: Supplementary file 1 [file cancers-15-04607-s001.zip › CATCA manuscript supplementary v01092023 clean version.pdf]

Analysis of plasma cell-free DNA genome-wide methylation in patients with colorectal cancer and advanced adenoma

Towards a cell-free DNA-based surveillance

Supplementary material

Table S1 Patient characteristics of patients included for cell-free DNA analyses.

ID	Sex	Location	Treatment	Age at diagnosis	Reason colonoscopy	Lesion	Characteristics‡	MSI status FFPE#
CATCA001	Male	Sigmoid	Laparoscopic sigmoid resection	57	SP	CRC	TxN0M0	MSS
CATCA006	Female	Transverse colon	Endoscopic mucosal resection	66	SP	AA	Tubulovillous adenoma (n=3), one ≥10 mm and one with high-grade dysplasia	MSS
CATCA008	Female	Hepatic flexure	Right hemicolectomy	73	SP	CRC	pT2N0	MSI-H
CATCA016	Male	Sigmoid	Neoadjuvant chemotherapy, sigmoid resection and wedge resection liver	67	SP	CRC	T3NxM1	MSS
CATCA036	Male	Sigmoid	Polypectomy	65	SP	AA	≥10 mm	MSS
CATCA038	Female	Transverse colon	Polypectomy	35	Lynch carrier, *MSH2*	AA	≥10 mm	MSI-H
CATCA044	Male	Rectum	Low-anterior resection	65	SP	CRC	pT3N2bM0	MSS
CATCA075	Female	Sigmoid	Polypectomy	68	SP	AA	Tubulovillous adenoma, ≥10 mm	MSS
CATCA099	Female	Sigmoid	Polypectomy	47	Lynch carrier, *PMS2*	AA	Tubulovillous adenoma, ≥10 mm	MSS
CATCA133	Female	Rectum	Polypectomy	59	SP	CRC	pT1	MSS

AA = advanced adenoma; CRC = colorectal cancer; SP = Dutch National Screening Program

Figure S1 cfDNA CNV Wisecondor profile of patient CATCA016. This patient had colorectal cancer with liver metastases.

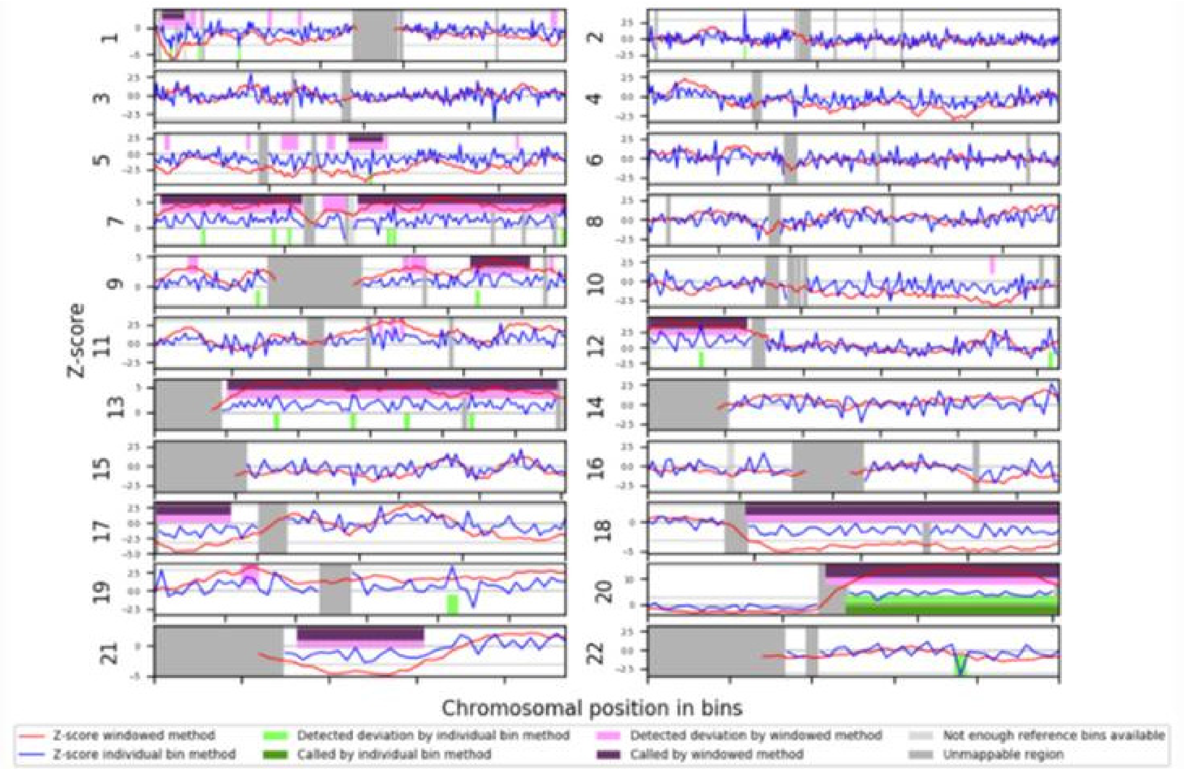

Figure S2 A) Median insert sizes and B) quantile 5% insert sizes in cfDNA of patients with advanced adenoma (green), colorectal cancer (blue), and controls (red).

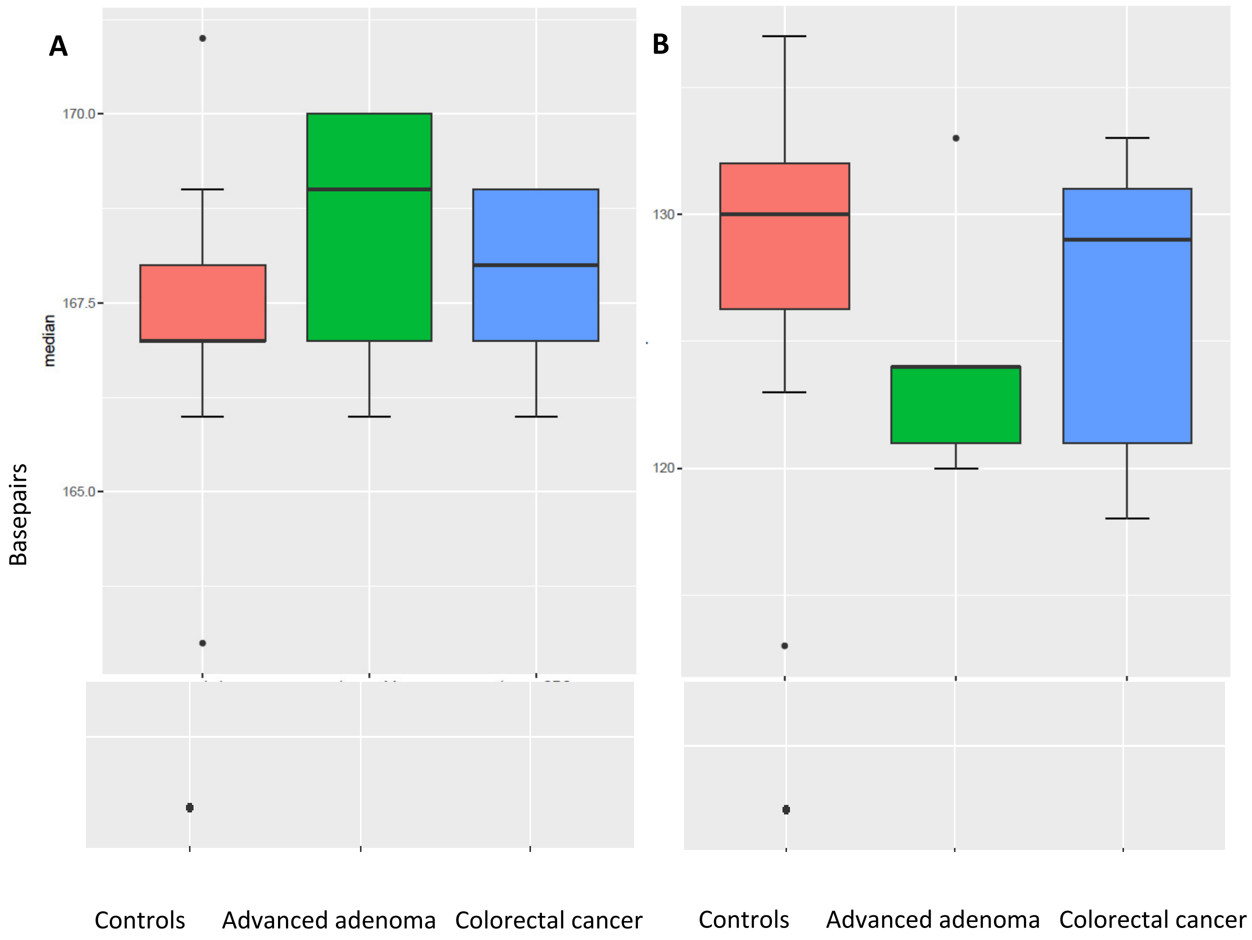

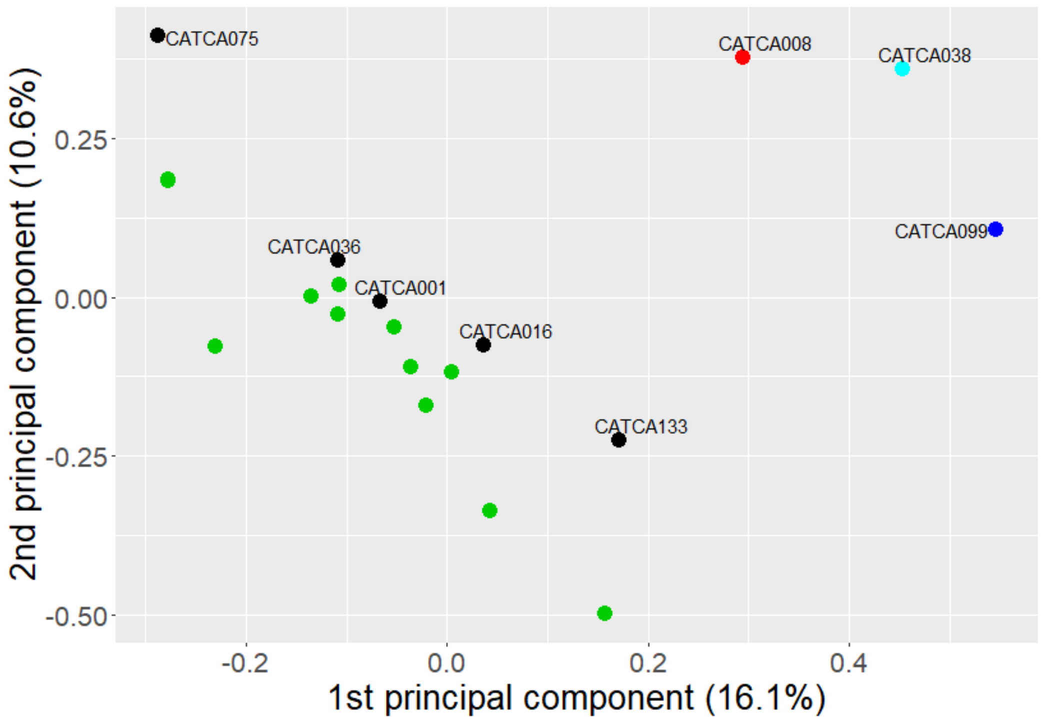

Figure S3 Principal Component Analysis (PCA) of patient samples analysed with genome-wide methylation analysis (MeD-seq) for patients included in our study and healthy blood donors (HBDs, in green), with on X and Y axis the percentage of variation that can be explained by that specific principal component. The patient with MSI-H CRC and LS carriers with AA are depicted in red and blue, respectively, while the MSS lesions are shown in black.

Table S3 Summary methylation scores upon genome-wide methylation analysis (MeD-seq), based on the entire genome (genome-wide summary score) or the 2798 differentially methylated regions (DMRs; DMR summary score) between patients and HBDs, for patients included in our study and healthy blood donors (HBDs). Genome-wide summary scores above 2.34 and DMR summary scores above 2.25 were considered elevated.

Sample ID	Sample type	Genome-wide summary score	DMR summary score
HBD1	Control	0.67	0.50
HBD2	Control	0.22	0.56
HBD4	Control	**2.52***	1.88
HDB5	Control	-1.09	-1.53
HBD7	Control	1.47	1.37
HBD19	Control	-0.99	-1.02
HBD20	Control	-0.92	-0.49
HBD21	Control	-0.35	-0.51
HBD22	Control	1.09	1.61
HBD24	Control	-1.15	-1.10
HBD25	Control	-0.46	-0.40
HBD29	Control	-0.49	-0.61
CATCA016	CRC, MSS	-0.74	**2.62***
CATCA001	CRC, MSS	-0.70	**2.62***
CATCA008	CRC, MSI-H sporadic	**17.42***	**582.50***
CATCA036	AA, MSS	-0.31	**5.65***
CATCA038	AA, Lynch syndrome	**6.93***	**202.65***
CATCA075	AA, MSS	2.20	**18.57***
CATCA099	AA, Lynch syndrome	**18.78***	**623.80***
CATCA133	CRC, MSS	-0.98	1.29
	**95% CI HBDs cut-off**	2.34	2.25

* Depicts elevated scores.
